# Traditional Chinese medicine-based visual assessment of tongue features associated with current cancer vs. no cancer history: a matched case-control study

**DOI:** 10.3389/fmed.2026.1761943

**Published:** 2026-01-30

**Authors:** Maria Benxia Wu, Stella Stylianou, Yong-Kang Wu, Benjamin Kimble, Brian May, Vincent Chan, Meng-Hua Chen, Suzi Mansu, Lin Dong, Byeongsang Oh, Daniel Man-yuen Sze, Zhen Zheng

**Affiliations:** 1 School of Health and Biomedical Sciences, STEM College, RMIT University, Bundoora, VIC, Australia; 2 School of Science, STEM College, RMIT University, Melbourne, VIC, Australia; 3 School of Computer Science, University of Sydney, Sydney, NSW, Australia; 4 School of Veterinary Science, University of Sydney, Sydney, NSW, Australia; 5 Aussway Chinese Medicine Centre, Melbourne, VIC, Australia; 6 Sydney Medical School, University of Sydney, Sydney, NSW, Australia; 7 Baptist Mission Australia, Sydney, NSW, Australia

**Keywords:** cancer survivorship, case-control study, sublingual veins, tongue inspection, traditional Chinese medicine, tongue image

## Abstract

**Background:**

Cancer survivorship is increasing, and many people living with and beyond cancer seek supportive care such as Traditional Chinese Medicine (TCM), where tongue inspection is routinely used. This exploratory study examined whether visually assessed tongue features differ between cancer survivors and those without a self-reported history of cancer.

**Methods:**

Matched case–control (*N* = 120): 60 adults diagnosed with cancer vs. 60 without a self-reported cancer history. Three standardized tongue photographs were taken for each participant. Three blinded TCM practitioners scored 20 predefined features. Logistic models with Benjamini–Hochberg false discovery rate (FDR) control estimated associations; Fleiss’ κ quantified inter-rater reliability. Nested logistic models explored discrimination; 10-fold cross-validation assessed area under the curve (AUC) and calibration.

**Results:**

Six features were found to be associated with cancer presence with an FDR of *q* < 0.05: peeled coating (odds ratio (OR) = 3.66, 95% confidence intervals (CI) 1.40–9.60), spider sublingual veins (OR = 2.74, 95% CI 1.53–4.92), thick coating (OR = 2.56, 95% CI 1.50–4.36), purple body (OR = 2.29, 95% CI 1.44–3.63), tooth marks (OR = 1.87, 95% CI 1.19–2.95), and thin coating (OR = 0.38, 95% CI 0.22–0.65; inverse). Reliability was slight to moderate (κ ≈ 0.01–0.48). Discrimination was modest (AUC 0.676–0.736).

**Conclusion:**

Selected tongue features, particularly spider sublingual veins and tooth marks, were associated with current cancer in exploratory analyses. However, discrimination was modest, and inter-rater reliability varied across features. These findings should be interpreted cautiously and do not support diagnostic utility. Standardized definitions, rater training, and external validation in larger prospective, multi-center cohorts are required before clinical translation.

## 1 Introduction

Cancer imposes a substantial and mounting health and economic burden in Australia and globally (1, 2). As advances in treatment options continue to extend survival, cancer is increasingly managed as a chronic condition rather than a single acute event (3). Individuals who live with cancer are considered cancer survivors for the remainder of their lives from the moment of diagnosis, regardless of active treatment, disease stability, or remission (4). Many survivorship studies describe persistent unmet supportive care needs across informational, physical, psychological, and practical domains, particularly after completion of first-line treatment (5, 6). They note feelings of abandonment when specialist-led care diminishes (5, 6). In this context, many people living with and beyond cancer seek supportive or integrative care options alongside conventional oncology. This includes Traditional Chinese Medicine (TCM) and other complementary therapies to manage ongoing symptoms, improve quality of life, and regain a sense of control in survivorship (5).

In TCM, clinical decision-making typically integrates patient-reported symptoms, palpation and direct visual observation. Tongue inspection is regarded as a core observational method and is interpreted as a reflection of systemic physiological and pathological states (7). Previous studies have reported associations between various cancers and specific tongue features, including color changes, coating characteristics, swelling, cracks, tooth marks (scalloping), and red dots. This suggests that morphological alterations of the tongue may carry diagnostic or prognostic information (5, 6, 8–12). However, most of these studies have relied on photographic images analyzed by automated tongue imaging systems or other computer-assisted platforms, rather than the visual inspection that is predominant in TCM clinical practice (5, 6, 8–12). Practitioner-based visual inspection is subjective and may be vulnerable to inter-observer variability and inconsistent reproducibility. Reporting inter-rater reliability is therefore essential when evaluating TCM assessment procedures. Prior studies in TCM, including tongue inspection, have reported variable inter- and intra-rater agreement across specific features and settings (8, 12, 13). This literature underscores that even when an association is observed, limited reproducibility can constrain interpretation and impede clinical translation.

This retrospective matched case–control study was designed to address this uncertainty. We explored whether visually assessed tongue features, scored using a binary (present/absent) framework from standardized photographs acquired under controlled lighting, differed between cancer survivors and individuals without a self-reported history of cancer. Specifically, we (i) explored tongue features that differed between groups and (ii) quantified inter-rater reliability of these visual assessments under standardized viewing conditions.

## 2 Materials and methods

### 2.1 Ethics approval and consent

This study was approved by the Human Research Ethics Committee of RMIT University (Approval Nos. 105-19/22600 and 23398). All procedures were conducted in accordance with relevant guidelines and regulations and adhered to the principles of the Declaration of Helsinki. Written informed consent was obtained from all participants prior to inclusion in the final analysis. At the time of invitation, participants were informed that participation was voluntary, that they could withdraw at any time without affecting their care, and that their data would be anonymized in any reports and publications.

### 2.2 Study design and participants

**FIGURE 1:**
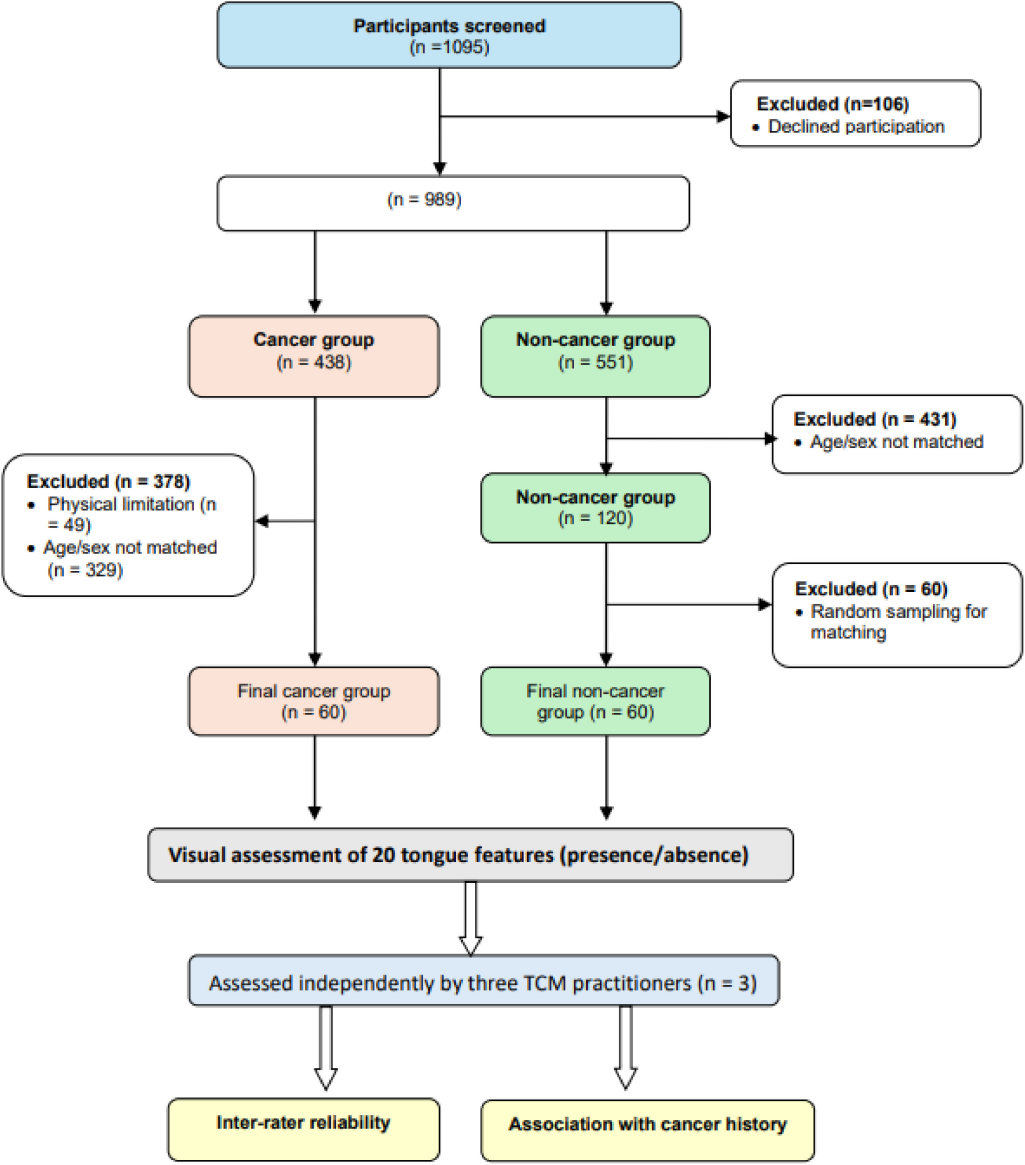
Study design and participant flow.

This matched case–control study was conducted at a private TCM clinic in Sydney, Australia. Between December 2019 and April 2023, all new and returning patients (*n* = 1,095 approached) were invited to participate; 106 declined and 989 were screened. Of these, 438 individuals self-reported a diagnosis of cancer and 551 reported no cancer history. Inclusion criteria were age ≥ 18 years, willingness to have their tongue photographed, and written consent for research use of de-identified images and records. Exclusions were applied for inadequate image capture and for lack of suitable age- or sex-matched counterparts. Sixty individuals with a history of cancer and 120 non-cancer participants consented. Non-cancer participants were then randomly subsampled to 60 to create a 1:1 matched dataset (age ± 1 year; sex). The final analytical sample comprised 120 participants (60 cancer survivors, 60 non-cancer) (Figure 1).

### 2.3 Development of the visual assessment protocol

Before commencing the case–control analysis, two pilot studies were conducted to optimize (i) how tongue features would be rated and (ii) how images would be acquired. These pilots provided information but were not included in the final dataset.

#### 2.3.1 Pilot study 1: scoring framework and inter-rater reliability

To determine an optimal rating method, 10 experienced TCM practitioners independently evaluated 60 tongue images using binary presence/absence scoring and semi-quantitative severity grades. Inter-rater reliability favored binary scoring (mean Fleiss’ κ = 0.417 ± 0.175) over severity grades (0.302 ± 0.131; *p* < 0.001), thus the binary scoring system was adopted for the main study.

#### 2.3.2 Pilot study 2: lighting conditions

Twenty individuals had their tongues photographed under ambient and flash lighting to determine which conditions best revealed key tongue characteristics. Based on this assessment, a standardized three-image protocol was established, and each of the target features was prospectively mapped to the image type (dorsal ambient lighting, dorsal flash, or ventral flash) that yielded optimal visibility.

### 2.4 Tongue image acquisition

Images were captured using the rear wide camera of an iPhone 11 Pro (12 MP, f/1.8; True Tone). For each participant, three images of the following tongue surfaces were obtained: (i) dorsal under ambient illumination, (ii) dorsal with flash, and (iii) ventral with flash. This configuration maximized the visibility of tongue body color, tongue coating, and sublingual vasculature.

### 2.5 Visual assessment and feature selection

A booklet was produced that presented each participant’s three images in fixed order with binary tick-boxes for 20 predefined tongue features.

### 2.6 Feature selection rationale

**FIGURE 2:**
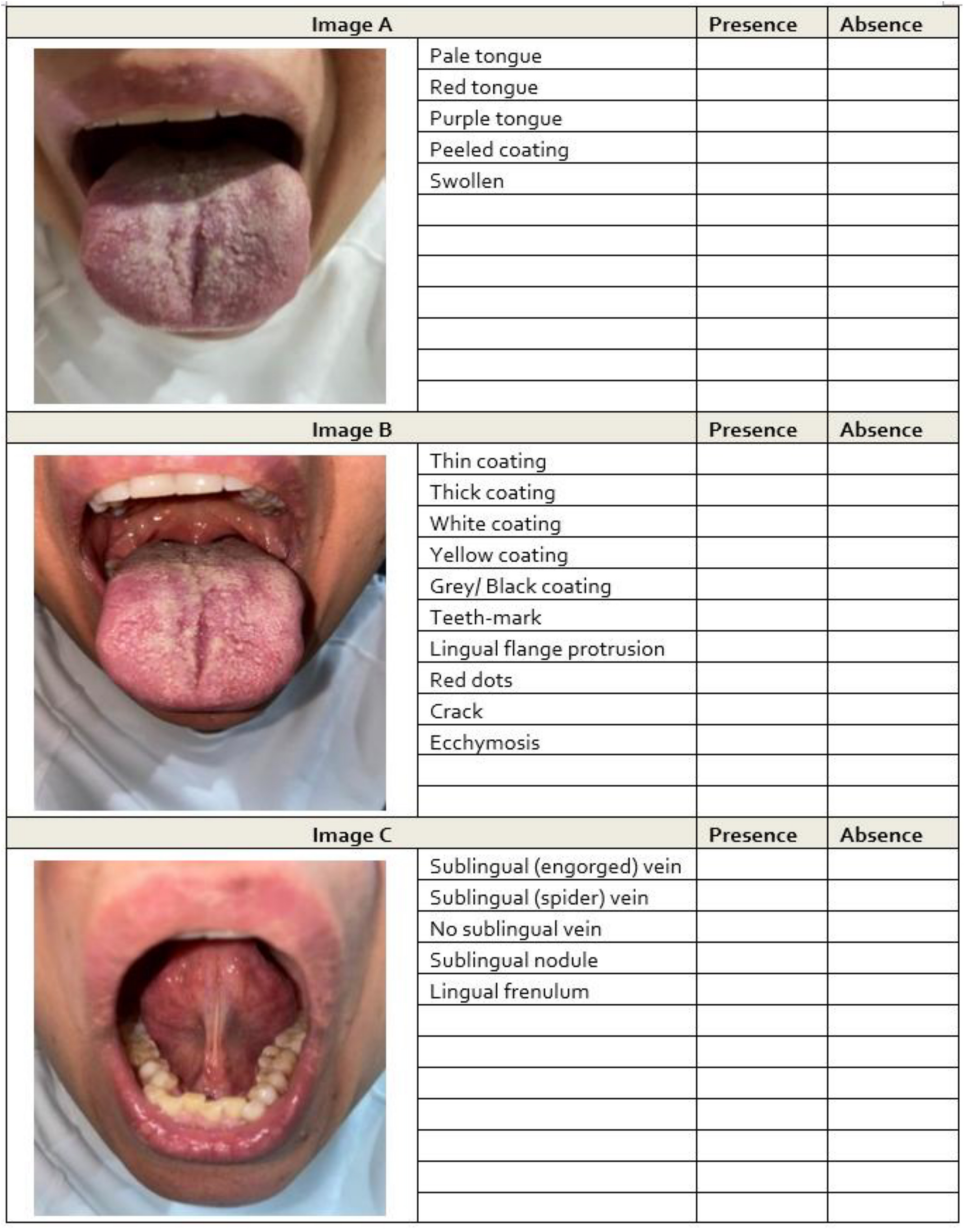
Example images of tongue features from participants. For each participant, three images were captured: **(A)** dorsal surface under natural light, **(B)** dorsal surface under flashed light, and **(C)** ventral surface under flashed light. Assessors independently evaluated the presence or absence of tongue features in each image. Feature identification was assigned to the optimal image type based on findings from the prior study.

The 20 features were selected a priori based on the standardized TCM tongue inspection taxonomy and include tongue body color (e.g., pale, red, purple), tongue coating color and texture (e.g., white, yellow, thick, thin, peeled), morphological indicators (e.g., swollen, tooth marks, cracks), and sublingual venous patterns (e.g., engorged, spider-like). Operational definitions were refined through the pilot work and standard TCM diagnostic references (Figure 2). Three expert TCM practitioners (>20 years of experience) who were registered with the Australian Health Practitioner Regulation Agency independently scored the booklet while blinded to all clinical information. All images were printed with uniform color management to ensure consistent visual assessment.

### 2.7 Statistical analysis

#### 2.7.1 Feature-level associations

Binary logistic regression was used to evaluate whether each tongue feature (present or absent) was associated with a history of cancer. Models included the rater as a categorical factor to account for systematic differences among assessors. For each feature, we reported odds ratios (ORs) with 95% confidence intervals (CIs), Wald χ^2^ statistics, and *p*-values. OR > 1.0 indicates higher odds of the feature being present among participants with a cancer history, and OR < 1.0 indicates lower odds (14).

#### 2.7.2 Predictive modeling

Predictive modeling was included to assess whether combinations of tongue features offer practical discriminative value beyond individual feature associations, which cannot be evaluated through OR alone. Using consensus labels (feature present in ≥ 2 of 3 assessments), we constructed three nested logistic regression models to classify cancer history: Model A (spider sublingual veins ++ peeled coating + purple tongue body), Model B (Model A + tooth marks + gray/black coating + thick coating), and Model C (Model B + sublingual nodule + swollen tongue).

#### 2.7.3 Internal validation and discrimination

To reduce optimism, 10-fold stratified cross-validation was used and out-of-fold predictions were pooled. Discrimination was summarized using the area under the receiver operating characteristic curve (AUC) with 95% CIs estimated from 1,500 bootstrap resamples of the pooled out-of-fold predictions.

#### 2.7.4 Calibration

Calibration was assessed using decile-wise observed versus predicted plots and logistic recalibration models estimating the intercept (α) and slope (β) with Wald 95% CIs.

#### 2.7.5 Inter-rater agreement

Agreement among the three assessors was quantified using percentage agreement and Fleiss’ κ with 95% Cis (15). They were interpreted according to Landis–Koch benchmarks (14, 15, 16).

#### 2.7.6 Multiple testing control

Twenty feature-wise tests were conducted, and the false discovery rate (FDR) was controlled using the Benjamini–Hochberg procedure. FDR-adjusted q-values are presented alongside raw *p*-values.

#### 2.7.7 Sensitivity analyses

Robustness of results were assessed via: (i) consensus-label regressions without rater terms; (ii) mixed-effects logistic regression with participant-level random intercepts; (iii) leave-one-assessor-out analyses; (iv) conditional logistic regression accounting for matched pairs; (v) restriction to features with at least moderate agreement (κ ≥ 0.40); (vi) multivariable models of jointly significant features using Firth penalization; (vii) alternative multiple-testing adjustments (Holm–Bonferroni, Benjamini–Yekutieli); and (viii) population-average Generalized Estimating Equations models with robust standard errors. Extended analyses are reported in Supplementary Methods.

#### 2.7.8 Software and reproducibility

Analyses were initially performed in IBM SPSS Statistics version 29, Microsoft Excel (Real Statistics Resource Pack), and GraphPad Prism (v3). To meet current reproducibility standards, all analyses and figure generation were re-implemented in R (v4.3.2) using scripted workflows (packages: *lme4*, *geepack*, *logistf*, *pROC*, *rms*, *DescTools*, *dcurves*/*rmda*, *irr*, *irrCAC*, *ggplot2*). The R code reproduces all reported results and is accompanied by a renv lockfile and Dockerfile to ensure full environment reproducibility. All statistical tests were two-sided with significance level α = 0.05.

#### 2.7.9 Sample size

This feasibility-level study included 120 participants (60 cancer survivors, 60 non-cancer), which provides > 80% power to detect moderate-to-large associations between individual tongue features and cancer history (odds ratios ≈ 2–3 at mid-range prevalences). For model discrimination (AUC ≈ 0.75), this sample yields an estimated standard error of ∼ 0.04–0.05 and is adequate for characterizing moderate effects but insufficient to reliably detect small differences between models. Accordingly, the sample size is appropriate for exploratory feature identification and pilot predictive model development. Larger cohorts are required for confirmatory validation.

## 3 Results

### 3.1 Participant characteristics

The matched case–control sample consisted of 120 participants: 60 individuals with a history of cancer and 60 without a cancer history. By design, groups were balanced on sex (30 females and 30 males in each group) and closely matched on age [cancer survivors: 54 ± 15 years; non-cancer: 53 ± 15 years; median (IQR): 54 (45.8–65.0) versus 55 (44.0–63.5), respectively] (Table 1). There was no material difference in age between female and male participants (Supplementary Data). All cancer survivors in the analytic sample reported advanced disease at presentation, with stage III in 13/60 (21.7%) and stage IV in 47/60 (78.3%). Most cancer survivors reported prior conventional oncologic treatment: surgery in 55/60 (91.6%) and chemotherapy and/or radiotherapy in 57/60 (95.0%). Documented primary tumor sites among cancer survivors included breast, ovarian, colorectal/rectal, acute myeloid leukemia, lung, prostate, and other sites (Table 1).

**TABLE 1:** Demographic and clinical characteristics of participants with and without a history of cancer.

Characteristics		Cancer survivors (*n* = 60)	Non-cancer (*n* = 60)	Total (*N* = 120)	*p*-value
Age	Age [mean ± SD; median (IQR)]	54 ± 15; 54 [45.8–65.0]	53 ± 15; 55 [44.0–63.5]	54 ± 15; 54.0 [45.0–65.0]	0.580
Sex	Female	30 (50.0%)	30 (50.0%)	60 (50.0%)	0.855
	Male	30 (50.0%)	30 (50.0%)	60 (50.0%)	1.000
Tumor type*	Breast	19 (31.7%)	n/a	n/a	n/a
	Ovarian	17 (28.3%)	n/a	n/a	n/a
	Colorectal/rectal	6 (10%)	n/a	n/a	n/a
	AML	5 (8.3%)	n/a	n/a	n/a
	Lung	5 (8.3%)	n/a	n/a	n/a
	Prostate	5 (8.3%)	n/a	n/a	n/a
	Other	27(45%)	n/a	n/a	n/a
Cancer stage	Stage 3	13 (21.7%)	n/a	n/a	n/a
	Stage 4	47 (78.3%)	n/a	n/a	n/a
Treatment history	Surgery	55 (91.6%)	n/a	n/a	n/a
	Chemotherapy and/or radiotherapy	57 (95.0%)	n/a	n/a	n/a
Tongue features	Pale body	21 (35.0%)	18 (30.0%)	39 (32.5%)	0.559
	Red body	26 (43.3%)	28 (46.7%)	54 (45.0%)	0.714
	Purple body	30 (50.0%)	17 (28.3%)	47 (39.2%)	0.015
	Thin coating	42 (70.0%)	54 (90.0%)	96 (80.0%)	0.006
	Thick coating	23 (38.3%)	10 (16.7%)	33 (27.5%)	0.008
	Peeled coating	5 (8.3%)	1 (1.7%)	6 (5.0%)	0.207
	White coating	49 (81.7%)	51 (85.0%)	100 (83.3%)	0.624
	Yellow coating	18 (30.0%)	14 (23.3%)	32 (26.7%)	0.409
	Gray/black coating	1 (1.7%)	0 (0.0%)	1 (0.8%)	1.000
	Swollen tongue	31 (51.7%)	22 (36.7%)	53 (44.2%)	0.098
	Tooth marks	25 (41.7%)	17 (28.3%)	42 (35.0%)	0.126
	Lingual flange protrusion	23 (38.3%)	20 (33.3%)	43 (35.8%)	0.568
	Red dot(s)	21 (35.0%)	26 (43.3%)	47 (39.2%)	0.350
	Crack(s)	41 (68.3%)	39 (65.0%)	80 (66.7%)	0.699
	Ecchymosis	10 (16.7%)	2 (3.3%)	12 (10.0%)	0.029
	Engorged sublingual veins	39 (65.0%)	31 (51.7%)	70 (58.3%)	0.139
	Spider sublingual veins	16 (26.7%)	4 (6.7%)	20 (16.7%)	0.006
	Absent sublingual veins	4 (6.7%)	5 (8.3%)	9 (7.5%)	1.000
	Sublingual nodule(s)	6 (10.0%)	3 (5.0%)	9 (7.5%)	0.491
	Lingual frenulum presence	49 (81.7%)	55 (91.7%)	104 (86.7%)	0.107

Values are n (%) unless stated; age is mean ± SD and median (IQR). *P*-values reflect unadjusted group comparisons (Mann–Whitney U for age; Fisher’s exact or χ^2^ for categorical variables) and are provided for context only; primary inference derives from the regression models in §3.2. Consensus for tongue features was defined as present if ≥ 2 of 3 assessors marked presence. *Participants were survivors of advanced cancer (stage III–IV) from a single primary site with regional and/or distant spread.

As descriptive context for subsequent modeling, the prevalence (consensus labels, ≥ 2/3 raters) of selected tongue features were as follows across the full cohort: white coating 100/120 (83.3%), thin coating 96/120 (80.0%), cracks 80/120 (66.7%), lingual frenulum presence 104/120 (86.7%), swollen tongue 53/120 (44.2%), tooth marks 42/120 (35.0%), purple body 47/120 (39.2%), engorged sublingual veins 70/120 (58.3%), and spider sublingual veins 20/120 (16.7%); rarer features included peeled coating 6/120 (5.0%) and gray/black coating 1/120 (0.8%). These proportions are descriptive only; inferential associations adjusted for assessor and corrected for multiple testing are reported in 3.2. Association models predicted the presence of each feature from cancer history, whereas predictive models reversed the direction and predicted cancer history from a set of features.

### 3.2 Association between tongue features and cancer survivors, and inter-rater agreement

**FIGURE 3:**
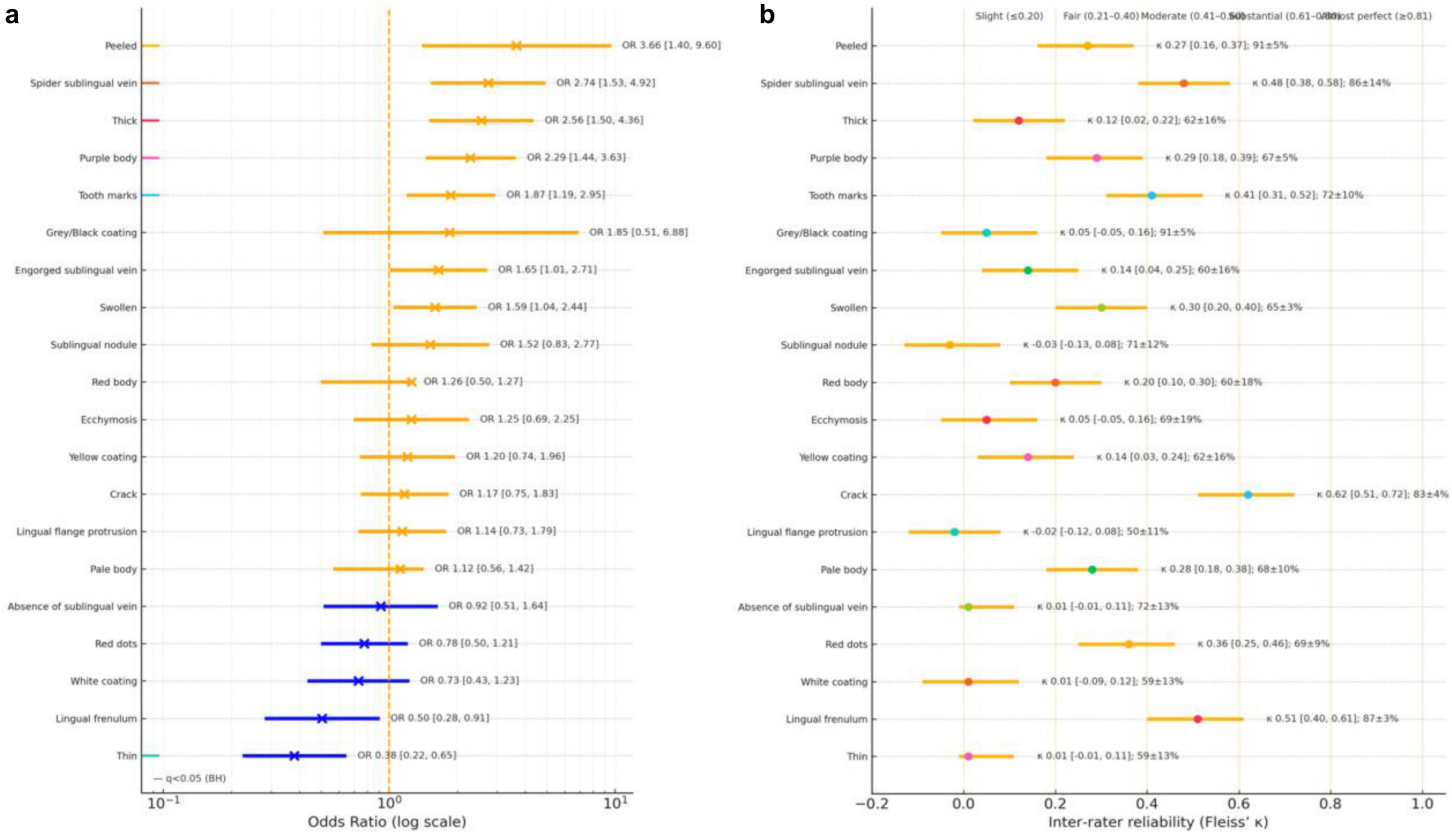
**(a,b)** Forest plot of odds ratios (95% confidence intervals) for cancer status as a predictor of each tongue feature (binary outcome: feature present vs. absent). Red bars indicate features more likely to be present in individuals with a history of cancer (OR > 1, 95% CI not crossing 1). Blue bars indicate features less likely to be present in individuals with a history of cancer (OR < 1, 95% CI not crossing 1). Gray bars indicate no statistically significant association.

We evaluated associations between visually assessed tongue features and cancer survivors as well as the consistency of these assessments across three expert TCM practitioners. ORs and 95% CIs were estimated from logistic models (feature present/absent ∼ cancer history + assessor). FDR-adjusted *q*-values (Benjamini–Hochberg across 20 tests) accompany raw p. Inter-rater reliability was quantified using Fleiss’ κ and percent agreement. A summary appears in Table 2; OR forest plots and κ plots are shown in Figures 3a,b.

**TABLE 2:** Logistic regression analysis on tongue features associated with cancer status and inter-rater reliability assessed with eye observation.

Tongue features		Logistic regression on tongue features associated with cancer status: Odd ratio (OR), 95% confidence interval (CI), *p*-value	Inter-rater reliability on tongue feature identification (presence/absence) by 3 TCM practitioners: Kappa (k), CI (lower, upper), Percentage of agreement
Dorsal surface	Colors	Pale body: OR = 1.118, 95% CI (0.563, 1.421), *p* = 0.637	*K* = 0.28, CI (0.18, 0.38); (Fair agreement); % agreement: 68 ± 10%
		Red body: OR = 1.258, 95% CI (0.497, 1.271), *p* = 0.339	*K* = 0.20 CI (0.10, 0.30); (Slight agreement) % agreement: 60 ± 18%
		Purple body: OR = 2.290, 95% CI (1.443, 3.634), *p* < 0.001*	*K* = 0.29, CI (0.18, 0.39); (Fair agreement) % agreement: 67 ± 5%
	Coating thickness	Thin: OR = 0.380, 95% CI (0.224, 0.646), *p* < 0.001*	*K* = 0.01, CI (-0.01, 0.11); (Slight agreement) % agreement: 59 ± 13%
		Thick: OR = 2.560, 95% CI (1.503, 4.359), *p* < 0.001*	*K* = 0.12, CI (0.02, 0.22); (Slight agreement) % agreement: 62 ± 16%
		Peeled: OR = 3.659, 95% CI (1.395, 9.599), *p* = 0.008*	*K* = 0.27, CI (0.16, 0.37); (Fair agreement) % agreement: 91 ± 5%
	Coating colors	White coating: OR = 0.730, 95% CI (0.434, 1.227), *p* = 0.235	*K* = 0.01, CI (-0.09, 0.12); (Slight agreement) % agreement: 59 ± 13%
		Yellow coating: OR = 1.203, 95% CI (0.739, 1.957), *p* = 0.457	*K* = 0.14, CI (0.03, 0.24); (Slight agreement) % agreement: 62 ± 16%
		Gray/Black coating: OR = 1.85, 95% CI (0.510, 6.88), *p* = 0.247	*K* = 0.05, CI (-0.05, 0.16); (Slight agreement) % agreement: 91 ± 5%
	Special tongue features	Swollen: OR = 1.595, 95% CI (1.042, 2.442), *p* = 0.032*	*K* = 0.30, CI (0.20, 0.40); (Fair agreement) % agreement: 65 ± 3%
		Tooth marks: OR = 1.874, 95% CI (1.191, 2.949), *p* = 0.007*	*K* = 0.41, CI (0.31, 0.52); (Moderate agreement); % agreement: 72 ± 10%
		Lingual flange protrusion: OR = 1.140, 95% CI (0.728, 1.786), *p* = 0.567	*K* = -0.02, CI (-0.12, 0.08); (No agreement) % agreement: 50 ± 11%
		Red dot(s): OR = 0.775, 95% CI (0.498, 1.207), *p* = 0.260)	*K* = 0.36, CI (0.25, 0.46); (Fair agreement) % agreement: 69 ± 9%
		Crack(s): OR = 1.169, 95% CI (0.747, 1.828), *p* = 0.494	*K* = 0.62, CI (0.51, 0.72); (Substantial agreement); % agreement: 83 ± 4%
		Ecchymosis: OR = 1.252, 95% CI (0.695, 2.255), *p* = 0.454	*K* = 0.05, CI (-0.05, 0.16); (Slight agreement) % agreement: 69 ± 19%
Ventral surface	Special tongue features	Engorged sublingual veins: OR = 1.653, 95% CI (1.008, 2.711), *p* = 0.046*	*K* = 0.14, CI (0.04, 0.25); (Slight agreement) % agreement: 60 ± 16%
		Spider sublingual veins: OR = 2.742, 95% CI (1.528, 4.920), *p* = 0.001*	*K* = 0.48, CI (0.38, 0.58); (Moderate agreement); % agreement: 86% ± 14%
		Absence of sublingual veins: OR = 0.916, 95% CI (0.511, 1.639), p = 0.088	*K* = 0.01, CI (-0.01, 0.11); (Slight agreement) % agreement: 72 ± 13%
		Sublingual nodule(s): OR = 1.518, 95% CI (0.832, 2.767), *p* = 0.173	*K* = -0.03, CI (-0.13, 0.08); (No agreement) % agreement: 71 ± 12%
		Lingual frenulum: OR = 0.504, 95% CI (0.280, 0.906), *p* = 0.022*	*K* = 0.51, CI (0.40, 0.61); (Moderate agreement); % agreement: 87 ± 3%

*significant difference (*p* < 0.05). Association between tongue features and cancer status (logistic regression) and inter-rater agreement (Fleiss’ kappa, % agreement) across three TCM practitioners. Significant associations with cancer status are indicated by *p < 0.05.

### 3.3 FDR-significant features

Six features remained associated with cancer survivors at *q* < 0.05: peeled coating (OR = 3.659, 95%CI 1.395–9.599), spider sublingual veins (OR = 2.742, 95%CI 1.528–4.920), thick coating (OR = 2.560, 95%CI 1.503–4.359), purple body (OR = 2.290, 95%CI 1.443–3.634), tooth marks (OR = 1.874, 95%CI 1.191–2.949), and thin coating (OR = 0.380, 95%CI 0.224–0.646; inverse association). Reliability varied from moderate (κ ≈ 0.48 for spider sublingual veins; κ ≈ 0.41 for tooth marks) to fair (κ ≈ 0.27–0.29 for peeled and purple) to slight (κ ≈ 0.12 for thick coating; κ ≈ 0.01 for thin coating).

### 3.4 Nominal but not FDR-significant

Three features had *p* < 0.05 but *q* ≥ 0.05: swollen tongue (OR = 1.595, 95%CI 1.042–2.442; p = 0.032; q≈0.08), lingual frenulum presence (OR = 0.504, 95%CI 0.280–0.906; *p* = 0.022; *q* ≈ 0.063; inverse), and engorged sublingual veins (OR = 1.653, 95%CI 1.008–2.711; *p* = 0.046; *q* ≈ 0.10). These trends warrant follow-up, particularly lingual frenulum presence, which had moderate reliability (κ = 0.51; 87% agreement).

Cancer history was associated with increased odds of purple tongue body (OR = 2.290, 95%CI 1.443–3.634; FDR-significant). Pale tongue body (OR = 1.118, 95%CI 0.563–1.421; *p* = 0.637) and red tongue body (OR = 1.258, 95%CI 0.497–1.271; *p* = 0.339) were not associated. Reliability for color categories was slight-to-fair (κ ≈ 0.20–0.29; 60–68% agreement).

#### 3.4.1 Tongue coating thickness (thin, thick, peeled)

Thick (OR = 2.560, 95%CI 1.503–4.359) and peeled coatings (OR = 3.659, 95%CI 1.395–9.599) were more likely in the cancer group, whereas thin coating was less likely (OR = 0.380, 95%CI 0.224–0.646). All three of them were FDR-significant but had different reproducibility: peeled tongue coating showed high raw agreement (91%) with fair κ (0.27), thick coating had 62% agreement with slight κ (0.12), and thin coating had 59% agreement with near-zero κ (0.01).

#### 3.4.2 Coating color (white, yellow, gray/black)

No coating color was associated after multiplicity control: white coating-color (OR = 0.730; *p* = 0.235), yellow (OR = 1.203; *p* = 0.457), and gray/black (OR = 1.85, 95%CI 0.51–6.88; *p* = 0.247). Reliability was generally slight (κ ≈ 0.01–0.14 for white/yellow; κ ≈ 0.05 for gray/black) despite moderate-to-high agreement in some cases, indicating limited beyond-chance concordance.

#### 3.4.3 Other dorsal tongue features (swelling, tooth marks, lingual flange protrusion, red dots, cracks, ecchymosis)

Tooth marks were associated with cancer history (OR = 1.874, 95%CI 1.191–2.949; FDR-significant) and had moderate agreement (κ = 0.41; 72%). Swelling of the tongue body (OR = 1.595, 95%CI 1.042–2.442; *p* = 0.032; q ≈ 0.08) showed fair reliability (κ = 0.30). Cracks in the dorsal tongue surface showed substantial agreement (κ = 0.62; 83%) but no association. Red dot(s) showed fair reliability (κ = 0.36) without association. Ecchymosis had slight agreement (κ = 0.05). Lingual flange protrusion had neither beyond-chance agreement (κ = –0.02) nor association.

#### 3.4.4 Ventral tongue features (sublingual vasculature and related findings)

Cancer history was associated with spider sublingual veins (OR = 2.742, 95%CI 1.528–4.920; FDR-significant; moderate κ = 0.48; 86%) and absence of a visible lingual frenulum (OR = 0.504, 95%CI 0.280–0.906; *p* = 0.022; q ≈ 0.063; moderate κ = 0.51; 87%). Engorged sublingual veins (OR = 1.653, 95%CI 1.008–2.711; *p* = 0.046; q ≈ 0.10) showed slight reliability (κ = 0.14). Sublingual nodules and absence of sublingual veins were not associated and showed no/slight agreement (κ = –0.03 and 0.01).

**FIGURE 4:**
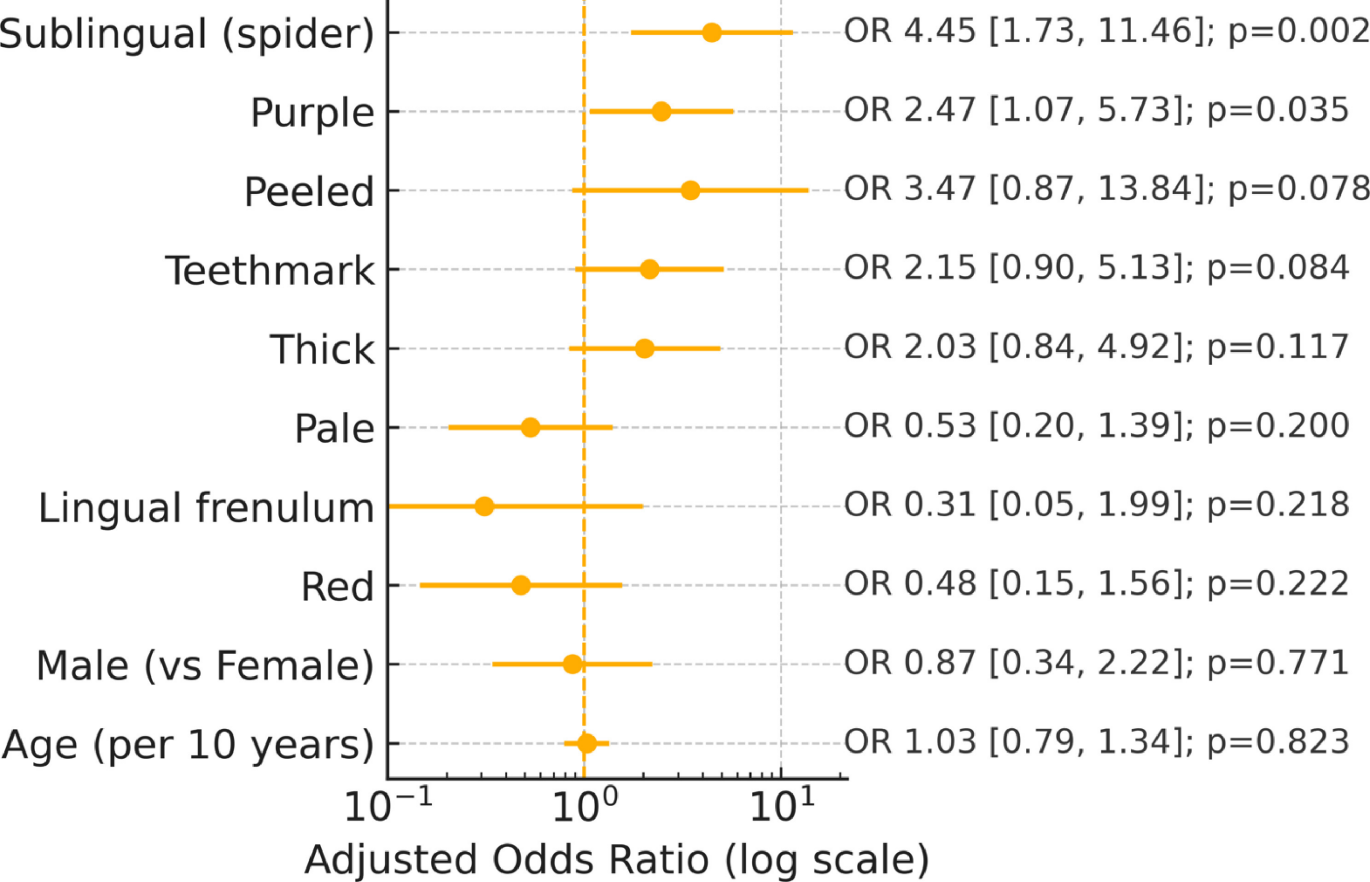
Adjusted predictor of cancer: Multivariable logistic regression (age/gender adjusted).

Features with both statistical support and at least moderate reliability (e.g., spider sublingual veins, tooth marks) represent the most credible candidates for validation. Signals with low κ (e.g., thin/thick coating) likely require rater calibration and harmonized definitions before clinical application (Figure 4).

### 3.5 Discrimination and calibration: modest predictive performance of tongue-feature models

**FIGURE 5:**
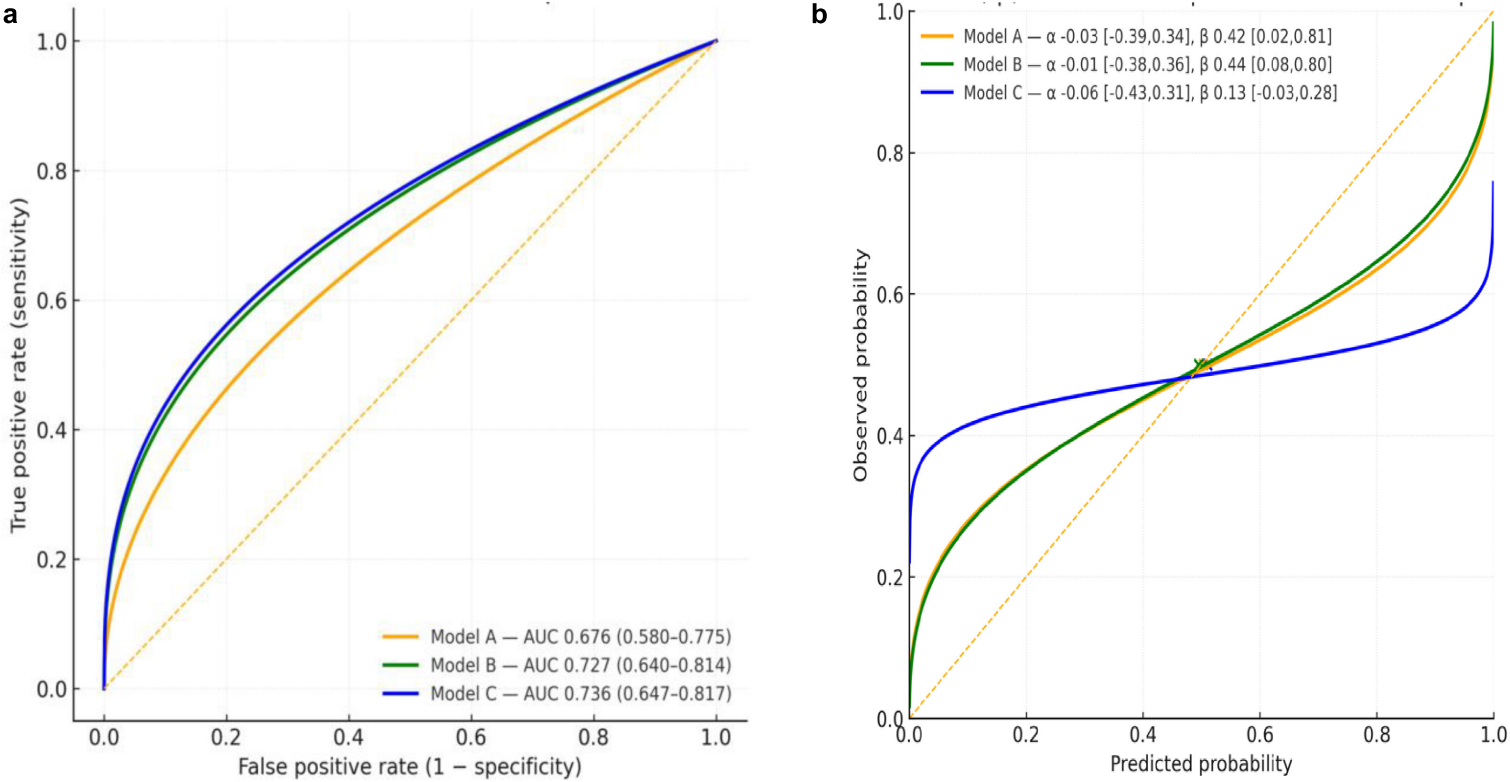
**(a)** ROC curves for Models A–C (10-fold CV, out-of-fold). Receiver operating characteristic curves with cross-validated AUCs (95%CI). Curves are parametric approximations matched to reported AUCs; empirical ROC curves can be provided from the model scores. **(b)** Calibration of Models A–C (10-fold CV). Calibration curves from logistic recalibration (observed vs. predicted). An intercept (α) near zero indicates calibration-in-the-large; slopes (β) < 1 indicate over-confidence. Points show the mean predicted risk against the observed prevalence (0.50).

Receiver operating characteristic (ROC) curves were generated for three nested logistic models using pooled out-of-fold predictions from 10-fold stratified cross-validation [Figure 5a (left): ROC curves]: Model A (spider sublingual veins, peeled coating, purple tongue body), Model B (Model A + tooth marks, gray/black coating, thick coating), and Model C (Model B + sublingual nodule, swollen tongue). Cross-validated AUCs (95% CI) were 0.676 (0.580–0.775), 0.727 (0.640–0.814), and 0.736 (0.647–0.817) for Models A–C, respectively. Models B and C improved discrimination over A across thresholds; the gain from B to C was small (difference in AUC ≈ 0.009), and overlapping CIs preclude a definitive separation by AUC alone.

Calibration-in-the-large was close to zero for all models: α_A = –0.03 (95% CI –0.39 to 0.34), α_B = –0.01 (–0.38 to 0.36) [Figure 5b (right): calibration curves], and α_C = –0.06 (–0.43 to 0.31). Calibration slopes were < 1, indicating overconfident probabilities with the greatest attenuation for Model C: β_A = 0.42 (0.02–0.81), β_B = 0.44 (0.08–0.80), and β_C = 0.13 (–0.03 to 0.28). Mean predicted risks (0.503, 0.495, and 0.510 for Models A–C respectively) closely matched the observed prevalence (0.50).

Overall, Model B provided the best balance between discrimination and calibration. Adoption of Model B with post hoc recalibration (e.g., slope adjustment) is reasonable pending external validation.

## 4 Discussion

In this sex- and age-matched case-control study of 120 adults (60 cancer survivors and 60 controls without a self-reported history of cancer), several practitioner-rated tongue features differed between groups. After adjusting for assessor effects and false-discovery rate, the results indicated that peeled tongue coating, spider sublingual veins, thick coating, a purple tongue body, and tooth marks were significantly more prevalent in the cancer group. Conversely, a thin tongue coating was less frequently observed. Due to the study’s exploratory nature and sample size, these associations warrant further investigation to confirm their diagnostic utility.

### 4.1 Interpretation of findings

The three predominant clusters of findings (i) color alteration (e.g., purple discoloration), (ii) surface/coating changes (peeled, thick, or scalloped coatings), and (iii) sublingual vascular changes are biologically plausible in the context of cancer and its treatment. Systemic malignancy and adjunct therapies may drive chronic inflammation, mucosal injury, anemia, salivary dysfunction, microbial dysbiosis, and microvascular remodeling (17–19). For example, tongue pallor may reflect anemia or impaired perfusion; thick or yellow coatings may signal altered salivary/epithelial turnover or biofilm accumulation (20–22); and prominent sublingual veins may mark venous stasis or microvascular adaptation phenomena that is also described in cardiovascular/metabolic disease (23–25). While none of these features are specific to cancer, their aggregation in the cancer cohort supports the notion of the tongue as a visible window into systemic physiological stress (26, 27).

### 4.2 Comparison to prior studies

Although empirical literature on systematic tongue feature assessment in cancer survivorship is limited, our findings align with two related evidence streams: oncology studies using computer-assisted or automated tongue analysis and broader work linking tongue coating and vascular patterns with metabolic or inflammatory states (12, 13, 28). Automated tongue inspection and image index studies in cancer populations (9–12, 29) report differences in tongue body color and coating characteristics that conceptually overlap our observations (e.g., purplish or discolored tongue body, coating thickness changes, and coating loss or peeling). Recent machine learning approaches (30, 31) further suggest tongue image phenotypes can carry a classification signal, although performance varies by cancer type, imaging protocol, and feature representation. Our study complements previous research by quantifying the reproducibility of visual tongue inspection. High consistency was found for features like spider sublingual veins and tooth marks; however, poor agreement for coating descriptors (*k* ≈ 0–0.12) suggests that observer variability may limit utility absent rigorous rater calibration and clearer definitions. Consistent with integrative oncology perspectives, our modest discrimination (AUC up to about 0.73) suggests tongue inspection is unlikely to be definitive alone but may provide adjunctive insight (32, 33).

### 4.3 Predictive values and limitations

Our nested multivariable models (Figure 5a,b) showed only moderate discrimination (Model A: AUC ≈ 0.68; Model B: ≈ 0.73; Model C: ≈ 0.74) with calibration slopes below 1. This indicates over-confident probability estimates, a hallmark of limited sample size and optimism bias (32, 33). These findings suggest that tongue inspection is insufficient as a standalone diagnostic tool; however, it may offer adjunctive utility, particularly for screening, triage, or identifying “red flags” within integrative care settings.

### 4.4 Inter-observer reliability and operationalization

Human raters in this study achieved moderate κ for features like spider sublingual veins (≈ 0.48) and tooth marks (≈ 0.41), but fair to slight reliability for more subjective traits (peeled coating ≈ 0.27; thin coating ≈ 0.01). These reliability metrics align with prior TCM tongue inspection literature (12, 13, 28) and underline the need for standardized photographic protocols, calibration training, and possibly automated image-analysis systems to increase reproducibility.

### 4.5 Clinical relevance

For the integrative oncology community, our findings raise two main potential applications. First, in the survivorship setting, tongue inspection is low-cost and non-invasive which could provide an early visual cue of mucosal, salivary, or vascular burden thereby prompting deeper evaluation in patients with a history of advanced cancer. Second, in resource limited settings, a structured tongue assessment tool might aid in early detection referral pathways; however, our data do *not* support using tongue features to diagnose cancer. Instead, they highlight that the oral cavity may reflect systemic disease and warrant further exploratory research in integrative cancer care.

### 4.6 Potential confounding factors

Many factors can influence tongue features: oral health and hygiene (34), smoking (35), alcohol (36), dentures (37), candidiasis (38), xerostomia/salivary dysfunction (39), medications (e.g., chemotherapy, steroids) (40), diabetes (41), cardiovascular disease (42), hydration, recent food/drink, and time of day (43). These factors were not systematically measured here and may confound observed associations. We therefore interpret results as associations, not effects. Future work should pre-specify an adjustment set (e.g., via a directed acyclic graph provided in Supplementary Figure 6), collect these covariates, and perform restriction and sensitivity analyses (e.g., excluding active oral infections, adjusting for xerostomia-inducing medications, stratifying by smoking).

### 4.7 Strengths and limitations

Key strengths were internal validity and measurement rigor. We used 1:1 age- and sex-matching to minimize confounding factors, standardized three-view image acquisition guided by pilot work, and a priori TCM feature definitions scored as present/absent. Three experienced, qualified TCM practitioners independently rated images while blinded to clinical information, and analyses accounted for assessor effects. We controlled multiplicity (FDR across 20 tests), quantified inter-rater agreement, and evaluated model performance with cross-validated discrimination and calibration. These measures support reproducible associations while transparently defining reliability limits for clinical translation. The limitations mirror those encountered in predictive biomarker research. Our cancer cohort consisted primarily of stage III/IV survivors, limiting inference about pre-diagnostic changes. We lacked stratification by cancer type, treatment modality, time since therapy, comorbidities, smoking status, or oral hygiene. Each of these was a potential confounding factor. Scoring was binary and subjective, and lighting, hydration, or tongue positioning may have influenced tongue appearance. Finally, the use of a single-center convenience sample limits generalizability. External validation in independent cohorts across multiple centers and diverse populations with standardized imaging conditions is needed to determine whether these associations and model performances generalize beyond our report.

### 4.8 Future research direction and conclusion

Prospective cohort studies that follow individuals without known cancer are needed to determine whether specific tongue changes predict future diagnosis or instead reflect treatment-related effects. Stratified analyses by cancer type, stage, treatment exposure and comorbidities will help clarify the biological relevance of each feature. Standardized high-resolution imaging combined with rater calibration or artificial intelligence -based analysis of color, texture, coating thickness, and vascular patterns may substantially improve measurement reliability and predictive accuracy (30, 31). As digital image analysis becomes increasingly integrated into clinical research, such work will be essential for establishing the prospective validity and clinical utility of TCM inspired tongue assessment in oncology settings.

In conclusion, we observed that several visually assessed tongue features differed between adults with cancer and those without a self-reported history of cancer. This combination of features provided only modest discriminative performance. Given the variable inter-rater reliability and inherent limitations of the matched case–control design, these findings should be interpreted as exploratory and hypothesis-generating. They support further structured and standardized investigation of tongue appearance as a potential adjunct assessment in TCM. Larger prospective, multi-center studies and external validation are required before any clinical translation.

## Data Availability

The raw data supporting the conclusions of this article will be made available by the authors, without undue reservation.
