# Supplementary material for: Traditional Chinese medicine-based visual assessment of tongue features associated with current cancer vs. no cancer history: a matched case-control study
Source: Front Med (Lausanne). 2026 Jan 30;13:1761943. doi: 10.3389/fmed.2026.1761943 (PMC12900746; doi:10.3389/fmed.2026.1761943)
Supplement: Supplementary file 1 [file Supplementary_file_1.docx]

**Supplementary Material**

**Statistical analysis**

**Association between tongue features and cancer history**

We used binary logistic regression to examine whether the presence of individual tongue features was associated with cancer history. For each tongue feature (dependent variable: 0 = absent, 1 = present), we modelled the effects of two independent variables: assessor (categorical; three assessors) and cancer status (0 = no history of cancer; 1 = history of cancer). Cancer status was the primary predictor of interest. This approach allowed us to estimate whether a given feature was more or less likely to be identified in individuals with a history of cancer, while accounting for systematic differences between assessors.

For each model, we report the regression coefficient (B), Wald χ² statistic and associated p-value, and the odds ratio [Exp(B)] with 95% confidence interval. In the context of this case–control design, the odds ratio represents the relative odds that a given feature is present in the cancer group compared with the non-cancer group (or vice versa), consistent with standard interpretation of odds ratios in observational studies. An odds ratio close to 1.0 indicates no detectable association between that feature and cancer status; an odds ratio >1.0 suggests a positive association (feature more common in individuals with a cancer history); and an odds ratio <1.0 suggests a negative association (41). In addition to feature-level associations, we conducted predictive modelling to determine whether combinations of TCM tongue features provide meaningful discriminative ability for identifying individuals with a cancer history. While odds ratios quantify association for each feature separately, ROC-based predictive models evaluate the overall classification performance of clinically motivated feature sets, which cannot be inferred from univariable analyses alone

**Predictive models**: In separate analyses from the feature-wise associations, we developed multivariable logistic regression models to classify cancer history (yes/no) from visually assessed tongue features (consensus labels; present if ≥2/3 raters). We evaluated three nested models: Model A (sublingual spider veins + peeled coating + purple tongue body), Model B: (Model A + tooth marks + grey/black coating + thick coating) and Model C: (Model B + sublingual nodule + swollen tongue).

**Internal validation and discrimination:** To mitigate optimism, we used 10‑fold stratified cross‑validation and pooled out‑of‑fold (OOF) predictions across folds. Discrimination was summarised by the area under the ROC curve (AUC) with 95% CIs obtained from 1,500 bootstrap resamples of the OOF predictions.

**Calibration:** Using the same OOF predictions, we assessed calibration via decile‑wise observed vs predicted plots and by estimating the calibration intercept (α) and slope (β) from a logistic recalibration model regressing the outcome on the logit of the predicted probabilities (Wald 95% CIs). Intercept values near 0 indicate calibration‑in‑the‑large; slopes <1 indicate over‑fitting/over‑confidence. We also report the mean predicted risk compared with the observed prevalence in this matched case–control sample (0.50).
**Inter-rater agreement**

For each feature we calculated percentage agreement—the proportion of cases where all three assessors recorded the same (present/absent)—and Fleiss’ κ with 95% CIs to quantify beyond‑chance agreement among three raters. κ was interpreted using Landis–Koch benchmarks: <0.00 poor, 0.00–0.20 slight, 0.21–0.40 fair, 0.41–0.60 moderate, 0.61–0.80 substantial, 0.81–1.00 almost perfect (42, 43).

Multiple testing control
Because 20 features were tested, we controlled the false discovery rate (FDR) using the Benjamini–Hochberg procedure across the 20 *p*‑values and report FDR‑adjusted q‑values alongside raw *p*. Unless specified, statistical significance is defined as q < 0.05.

## Sensitivity analyses

We conducted a prespecified set of robustness checks to evaluate whether the observed associations between tongue features and cancer history were sensitive to analytic choices and measurement variability.

1. **Consensus labels (primary alternative):** For each feature, presence was defined by majority vote across the three assessors (present if ≥2/3). We refit logistic regressions with feature presence as the outcome and cancer history as the predictor (no assessor term), reporting odds ratios (ORs) with 95% CIs and raw p‑values; Benjamini–Hochberg false discovery rate (FDR) control across the 20 features yielded q‑values.
2. **Mixed‑effects logistic regression (rater‑level data):** Treating each assessor’s rating as a repeated measure, we fitted generalised linear mixed models with a logit link, a random intercept for participant (to account for within‑participant clustering), cancer history as a fixed effect, and assessor as a fixed effect. This model estimates the association between cancer history and feature presence while accounting for correlation among the three ratings per participant.
3. **Leave‑one‑assessor‑out analysis:** To assess influence of individual raters, we repeated the main (fixed‑effects) logistic regression three times, each time excluding one assessor’s ratings, and compared the resulting ORs and q‑values.
4. **Matched‑pair analysis (design‑based):** Because a 1:1 age‑ and sex‑matched case–control set was constructed, we performed conditional logistic regression with case status as the outcome and each feature (consensus label) as the predictor, stratifying by matched pair. This analysis estimates pair‑conditioned odds ratios and serves as a design‑concordant robustness check. For comparability with the main analysis, we also present the reciprocal interpretation (feature odds given cancer history) in the Supplement.
5. **Restriction to reliably detected features.** To examine whether conclusions depended on measurement quality, we re‑estimated associations restricted to features with at least “moderate” agreement (Fleiss’ κ ≥ 0.40) and reported which signals persisted at q < 0.05. We also summarised results for κ ≥ 0.30 (“fair‑plus”) as a secondary threshold.
6. **Mutual adjustment among leading features**: To probe whether signals were independent, we constructed a multivariable logistic model including the set of features with q < 0.10 in the main analysis (to avoid over‑fitting given n = 120), with Firth penalisation when separation occurred. This analysis is considered exploratory and results are reported in the Supplement.
7. **Alternative multiple‑testing controls:** In addition to Benjamini–Hochberg FDR (main text), we applied Holm–Bonferroni (FWER) and Benjamini–Yekutieli (FDR under positive dependence) across the 20 tests; we report how the set of significant features changed, if at all, under these stricter procedures (Supplement).
8. **Robust SEs / GEE check**: As an alternative to the mixed‑effects approach, we fit population‑average GEE models with participant‑level clustering (exchangeable working correlation) and Huber–White robust SEs; estimates were compared to the main models.

**Software and reproducibility**
Analyses were initially performed in IBM SPSS Statistics, Microsoft Excel (Real Statistics Resource Pack), and GraphPad Prism (v3). To meet current reproducibility standards, all analyses and figure generation were re-implemented in R (v4.3.2) using scripted workflows (packages: *lme4*, *geepack*, *logistf*, *pROC*, *rms*, *DescTools*, *dcurves*/*rmda*, *irr*, *irrCAC*, *ggplot2*). The R code reproduces all reported results and is accompanied by a renv lockfile and Dockerfile to ensure full environment reproducibility. All statistical tests were two-sided with significance level α = 0.05.

**Supplementary Table S1. Tongue Feature Codebook (simplified)**

| **Tongue feature** | **Imaging view used** | **Operational definition (image-based)** |
| --- | --- | --- |
| Spider-like sublingual veins | Ventral flash | Stellate venous pattern on the ventral tongue with a visible central hub and ≥3 fine venous branches radiating outward, clearly distinct from a single linear or tortuous engorged vein. |
| Peeled coating | Dorsal ambient | One or more sharply demarcated areas of absent coating exposing smooth, glossy, erythematous mucosa; scored present if the cumulative peeled area ≥10% of the dorsal surface and not limited to fissures. |
| Tooth marks (scalloping) | Dorsal ambient | ≥2 visible indentations along at least one lateral tongue border, aligned with dental impressions and clearly distinguishable from irregular edge contours or motion artefact. |
| Thick coating | Dorsal ambient | Opaque or dense tongue coating that obscures underlying papillae over a substantial portion of the dorsal surface. |
| Thin coating | Dorsal ambient | Translucent coating through which papillae and surface texture remain clearly visible over most of the dorsal surface. |
| Purple tongue body | Dorsal ambient | Diffuse violaceous or purplish discoloration involving a substantial portion of the tongue body, persisting across lighting conditions. |

Scoring rule: Each feature was independently scored as present or absent by three blinded TCM practitioners. Participant-level presence was defined by consensus (≥2 of 3 raters).

**Table S3. Prevalence and association of FDR-significant tongue features with cancer history**

| **Tongue feature** | **Cancer group n/N (%)** | **Control group n/N (%)** | **Odds ratio (95% CI)** |
| --- | --- | --- | --- |
| Peeled coating | 5/60 (8.3%) | 1/60 (1.7%) | 3.66 (1.40–9.60) |
| Spider-like sublingual veins | 16/60 (26.7%) | 4/60 (6.7%) | 2.74 (1.53–4.92) |
| Thick coating | 23/60 (38.3%) | 10/60 (16.7%) | 2.56 (1.50–4.36) |
| Purple tongue body | 30/60 (50.0%) | 17/60 (28.3%) | 2.29 (1.44–3.63) |
| Tooth marks (scalloping) | 25/60 (41.7%) | 17/60 (28.3%) | 1.87 (1.19–2.95) |
| Thin coating (inverse association) | 42/60 (70.0%) | 54/60 (90.0%) | 0.38 (0.22–0.65) |

Odds ratios were estimated from logistic regression models adjusted for assessor. Features shown met FDR significance (q < 0.05).

**Supplementary Table S4. Sensitivity analysis using stricter reliability and consensus criteria**

| **Model** | **Primary analysis**  **(2/3 consensus) AUC** | **κ ≥ 0.40 only AUC** | **Consensus**  **(3/3) AUC** |
| --- | --- | --- | --- |
| Model A | 0.67 | 0.66 | 0.65 |
| Model B | 0.73 | 0.72 | 0.71 |
| Model C | 0.74 | 0.73 | 0.72 |

Primary analysis defined feature presence by agreement of at least 2 of 3 raters. The κ ≥ 0.40 analysis restricted predictors to features with at least moderate inter-rater reliability. The stricter consensus analysis required agreement by all three raters (3/3). Across sensitivity analyses, discrimination decreased slightly as expected, but model ranking was preserved (Model C ≳ Model B > Model A), supporting robustness of the primary findings.
